# Supplementary material for: Ultra-conformal skin electrodes with synergistically enhanced conductivity for long-time and low-motion artifact epidermal electrophysiology
Source: Nat Commun. 2021 Aug 12;12:4880. doi: 10.1038/s41467-021-25152-y (PMC8361161; doi:10.1038/s41467-021-25152-y)
Supplement: Supplementary file 1 — Supplementary Information [file 41467_2021_25152_MOESM1_ESM.docx]

**Ultra-Conformal Skin Electrodes with Synergistically Enhanced Conductivity for Long-Time and Low-Motion Artifact** **Epidermal Electrophysiology**

*Yan Zhao*^1^*, Song Zhang*^2^*, Tianhao Yu*^3^*, Yan Zhang*^1^*, Guo Ye*^1^*, Han Cui*^4, 5^*, Chengzhi He*^6^*, Wenchao Jiang*^3^*, Yu Zhai^7^, Chunming Lu^7^, Xiaodan Gu*^2^ *and Nan Liu*^1,3*^

^1^ *Beijing Key Laboratory of Energy Conversion and Storage Materials, College of Chemistry, Beijing Normal University, Beijing 100875, China*

^2^ *School of Polymer Science and Engineering, The University of Southern Mississippi, Center for Optoelectronic Materials and Device, Hattiesburg, Mississippi 39406, United States*

^3^ *Beijing Graphene Institute, Beijing 100094, China*

*^4^ Department of Acupuncture and Moxibustion, Shenzhen Traditional Chinese Medicine Hospital, Shenzhen, China*

*^5^ CAS Key Laboratory of Hu man-Machine Intelligence-Synergy Systems, Shenzhen Institutes of Advanced Technology, Chinese Academy of Sciences, Shenzhen, China,*

^6^ *Beijing Advanced Innovation Center for Soft Matter Science and Engineering, Beijing University of Chemical Technology, Beijing 100029, China*

*^7^ State Key Laboratory of Cognitive Neuroscience and Learning & IDG/McGovern Institute for Brain Research, Beijing Normal University, Beijing 100875, China*

* Corresponding author: E-mail: nanliu@bnu.edu.cn


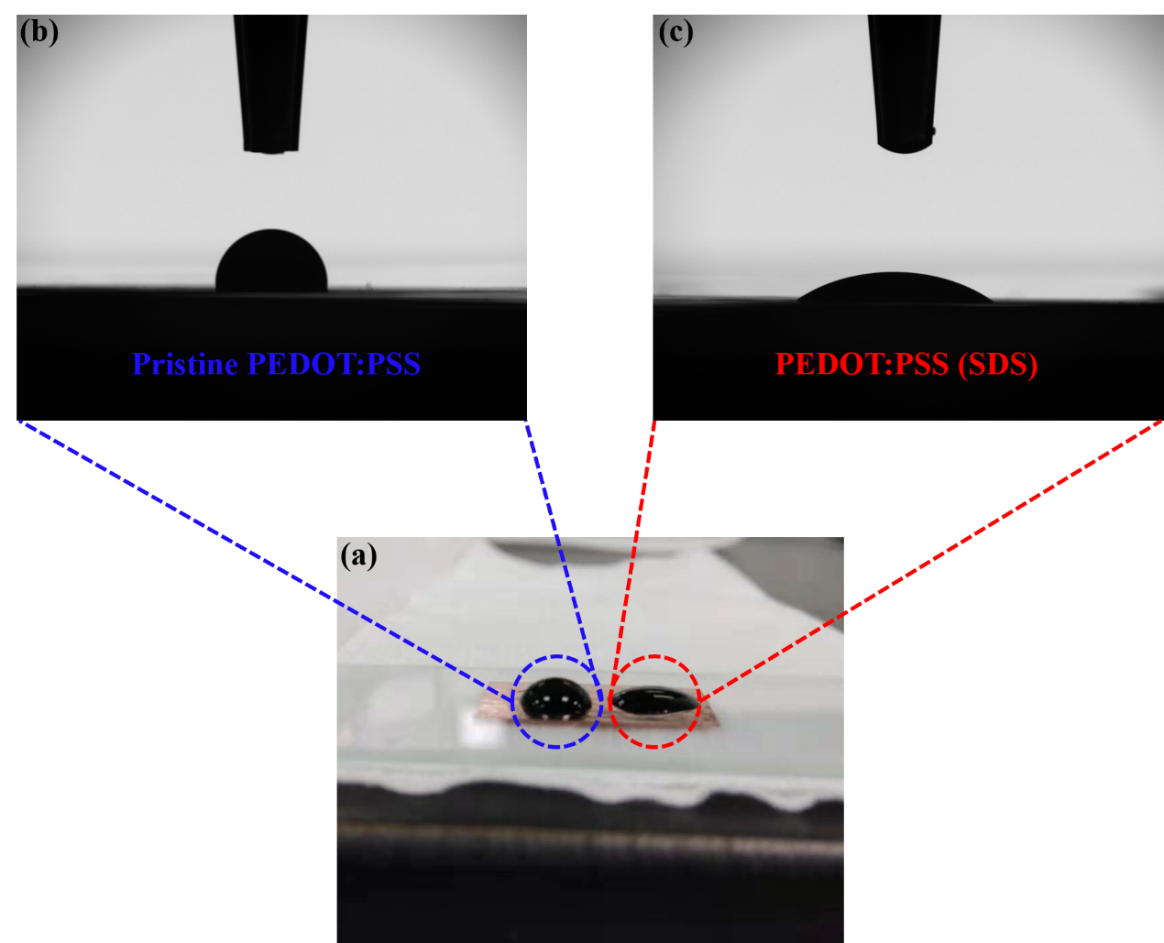


**Supplementary Figure 1** Contact angle between PEDOT:PSS and graphene, images of PEDOT:PSS on graphene with copper (a), where (b) was pristine PEDOT:PSS and (c) was PEDOT:PSS with SDS.


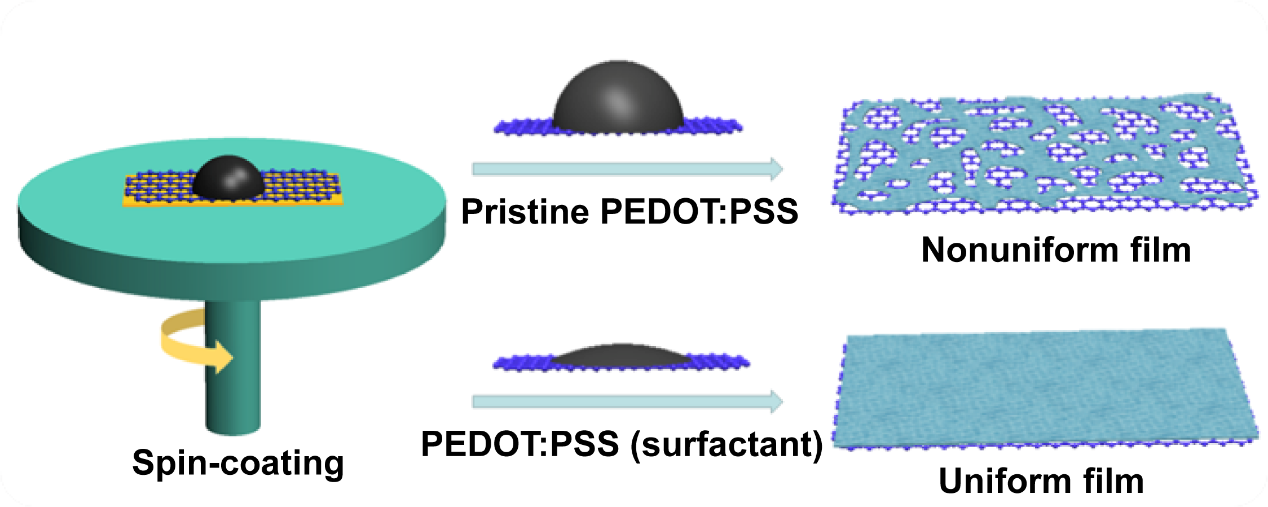


**Supplementary Figure 2** Schematic illustration of the fabrication process of continuous and in-continuous PEDOT:PSS on graphene, showing the function of surfactants.


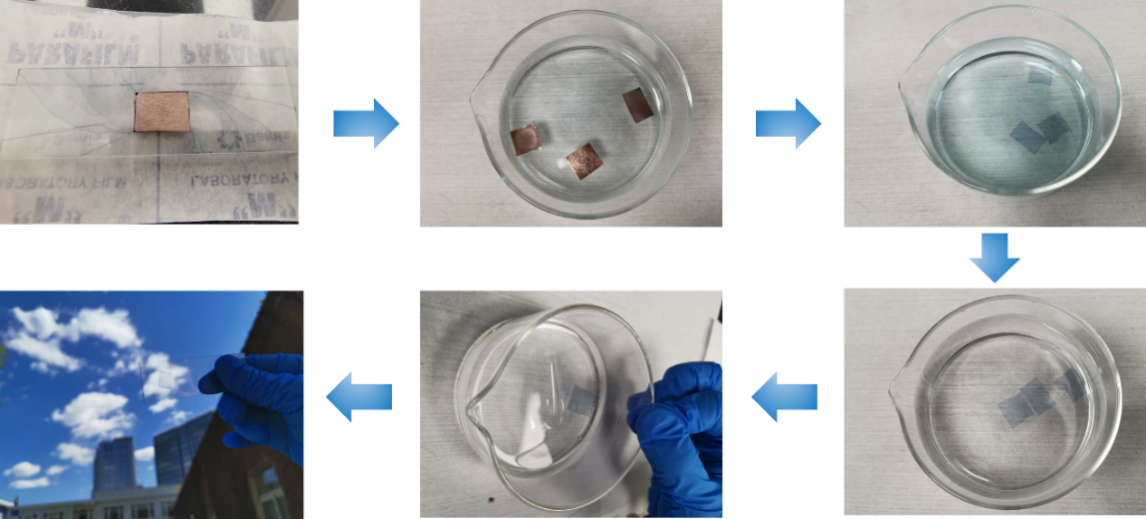


**Supplementary Figure 3** Pictures shows the procedures of Cu etching and transfer to obtain PTG on glass.

After spin-coating and annealing, the sample was floated in (NH_4_)_2_S_2_O_8_ solution to etch the underlying Cu foil, followed by rinsing in deionized water to remove residues. The resultant PTG thin film can be scooped onto an arbitrary substrate. The PTG on glass was quite transparent.


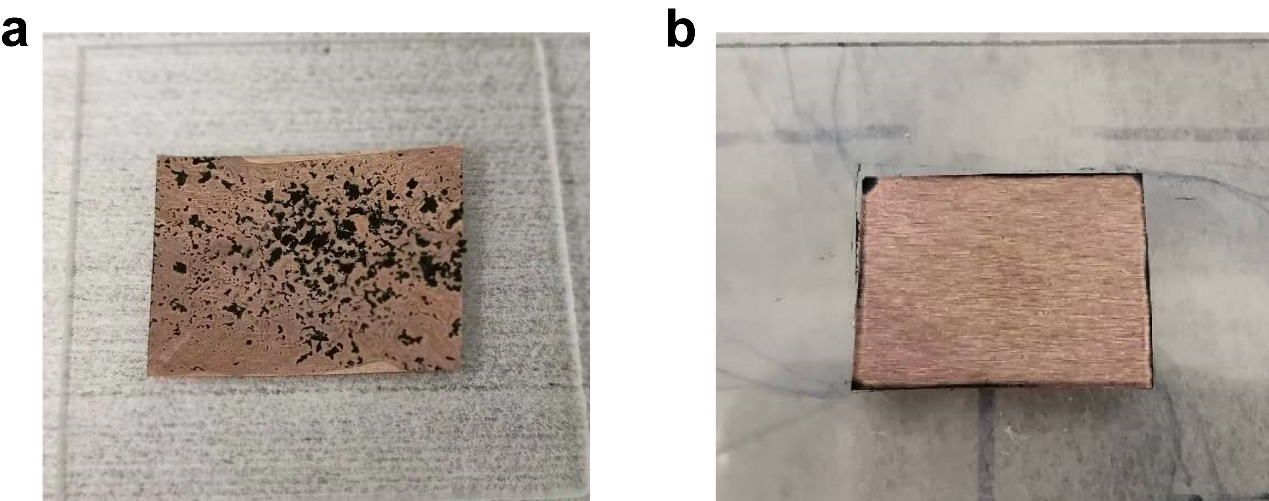


**Supplementary Figure 4** The pictures of (a) precipitated PEDOT:PSS on graphene film and (b) uniform PTG.


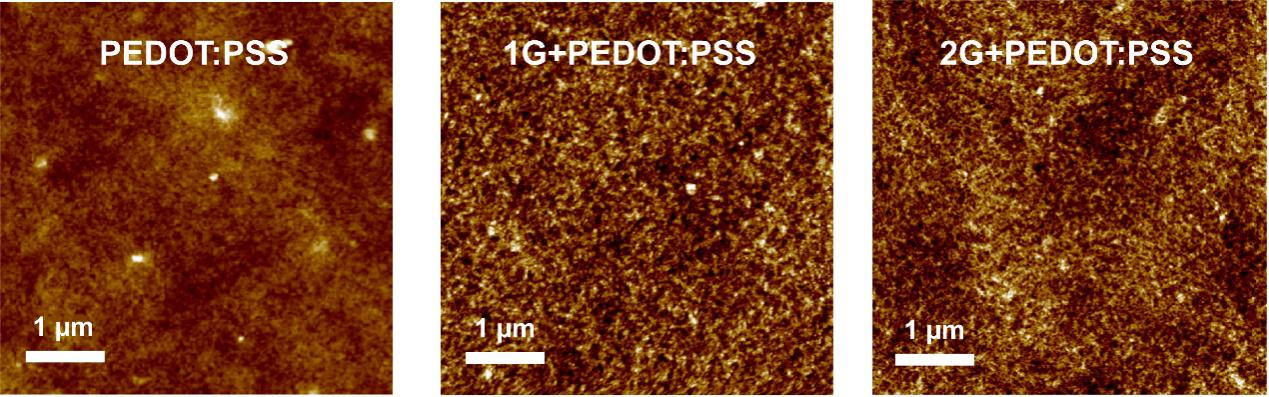


**Supplementary Figure 5** Morphological comparison of pure PEDOT:PSS and PTGs (mono- and bi-layer graphene) on SEBS by AFM.


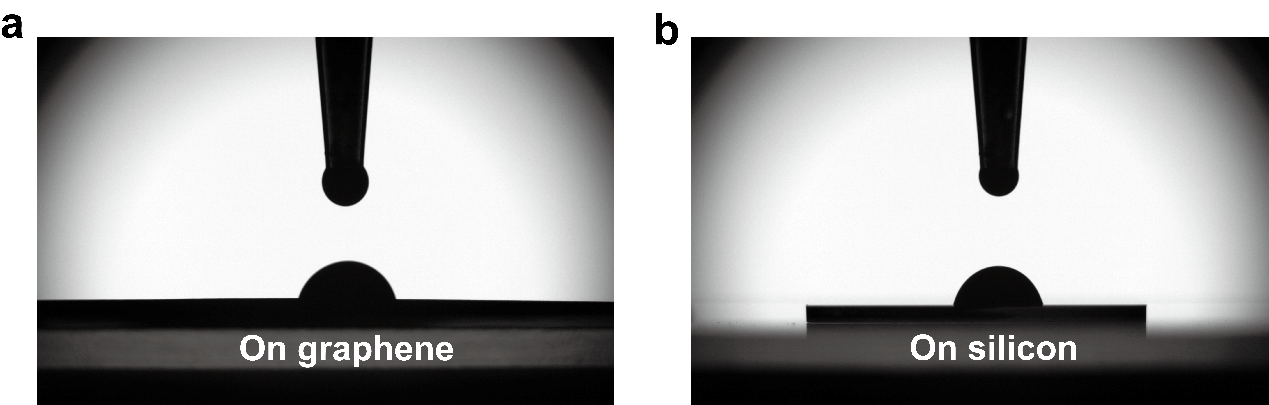


**Supplementary Figure 6** Contact angle of PEDOT:PSS on graphene (a) and silicon (b)


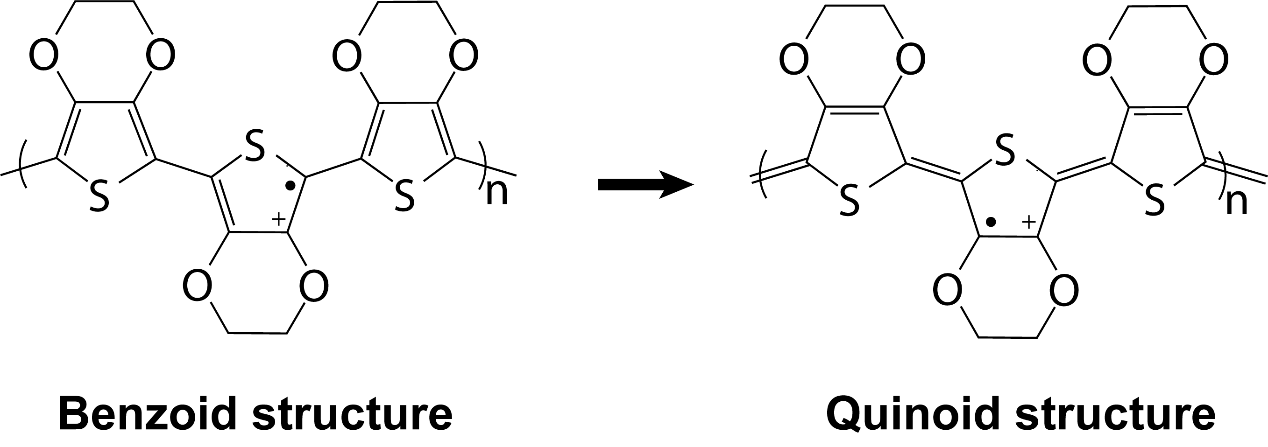


**Supplementary Figure 7** The structure transition of PEDOT chain from benzoid to quinoid structure.


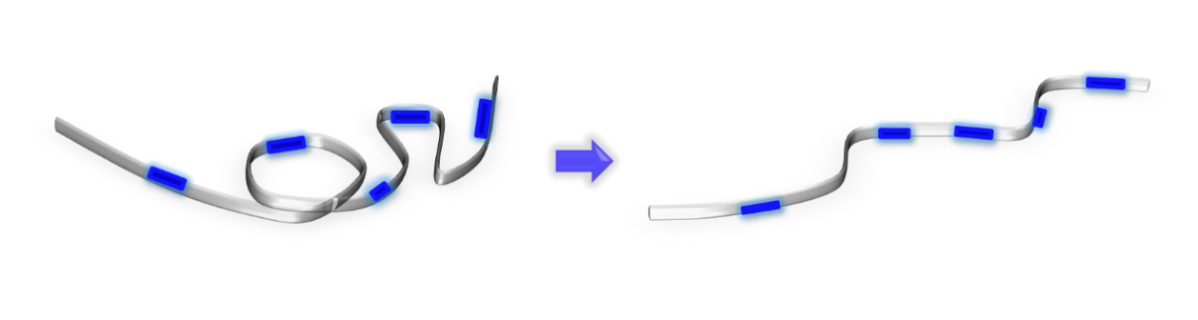


**Supplementary Figure 8** The structure transition of PEDOT:PSS from coil to linear. The thiophene ring on PEDOT chains transits from a coil-like benzoid structure in pristine to a linear-like quinoid structure. With the addition of SDS and BSL, the coiled PEDOT chain in the pristine PEDOT:PSS aqueous solution became linear.


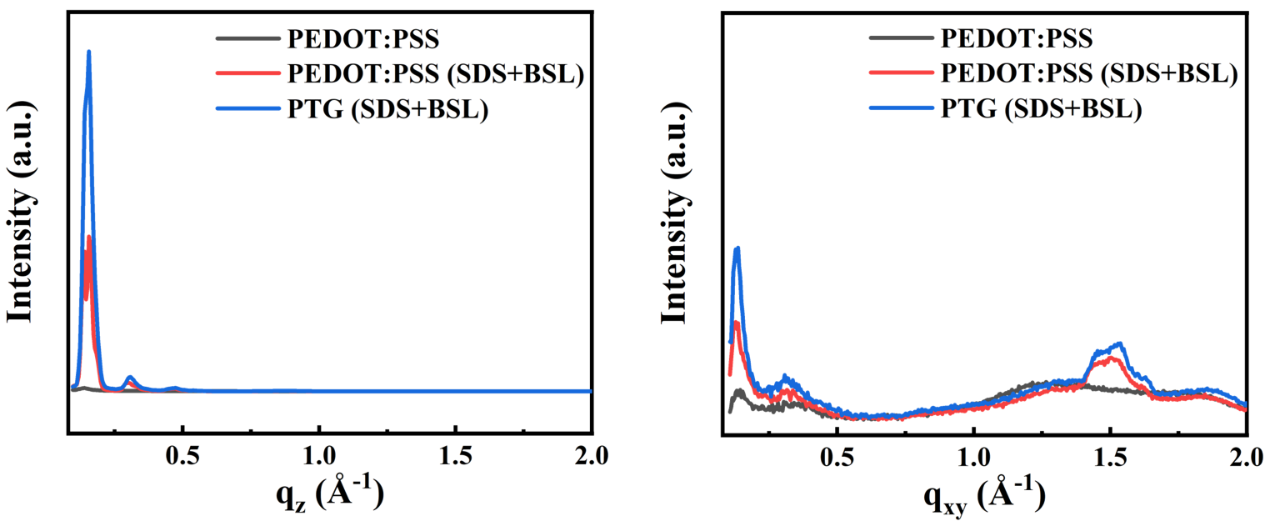


**Supplementary Figure 9** The 1D scattering q_z_ and q_xy_ profiles obtained from the 2D GIWAXS patterns.


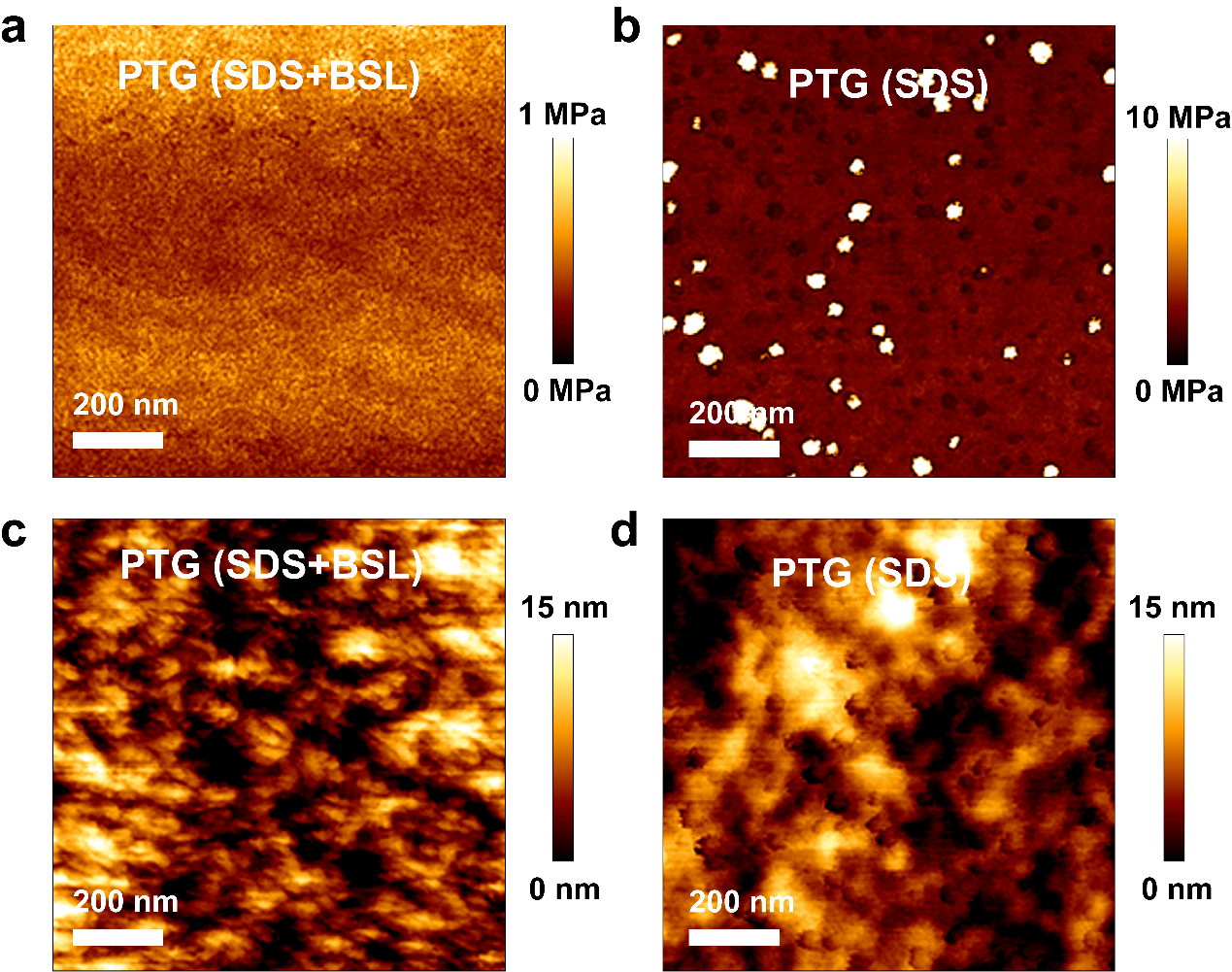


**Supplementary Figure 10** AFM height images and Young´s modulus images of PTG with BSL (a, c) and without BSL (b, d).

**Discussion about bending stiffness of PTG and skin:**

To interpret the stable attachment of PTG electrodes on skin during electrophysiological measurement, we introduced the concept of bending stiffness. Bending stiffness refers to the amount of stiffness that the subject will deflect. The smaller the stiffness, the larger the deflection will occur. According to the formula (1) and (2), bending stiffness ($\overline{{EI}_{sum}}$) of our PTG electrode can be calculated as following:

$\overline{EI}= \frac{1}{12 (1-\upsilon^{2})}\overline{E}h^{3}$ (1)

$\overline{{EI}_{sum}}= \alpha\overline{{EI}_{electrode}}+(1-\alpha) \overline{{EI}_{substrate}}$ (2)

Where $\overline{E}$ represents Young’s modulus, h represents thickness, $\upsilon$ represents Poisson ratio, and $\alpha$ represents the area ratio of electrode to substrate. Together with the small Young’s modulus and ultrathin nature, bending stiffness of our PTG electrode (with tattoo substrate) is ~ 0.00236 pNm. Correspondingly, according to above formular, bending stiffness of skin is ~ 0.00156 pNm. (Young’s modulus of skin is ~150 kPa, and the thickness of stratum corneum is ~ 15 μm)^1^. They match with each other very well. The matched bending stiffness leads to the ultra-conformability between PTG electrodes and skin. Once two subjects form ultra-conformability with each other, they tend to attach intimately. Besides, the existence of sweat may introduce capillary force, which can enhance the intimate contact and stable attachment^2^.


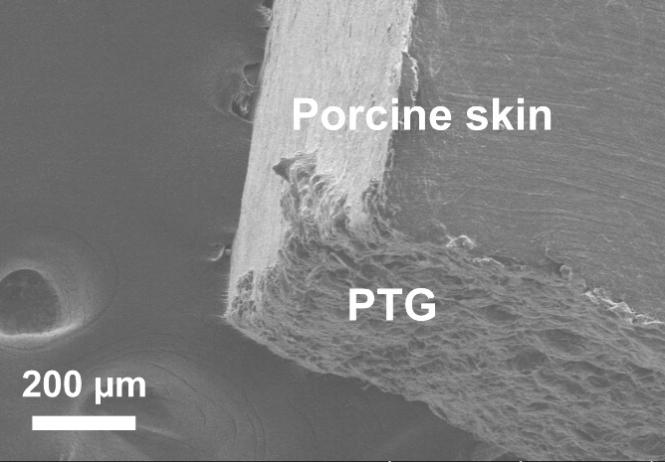


**Supplementary Figure 11** SEM image of PTG (bottom) on porcine skin (top).


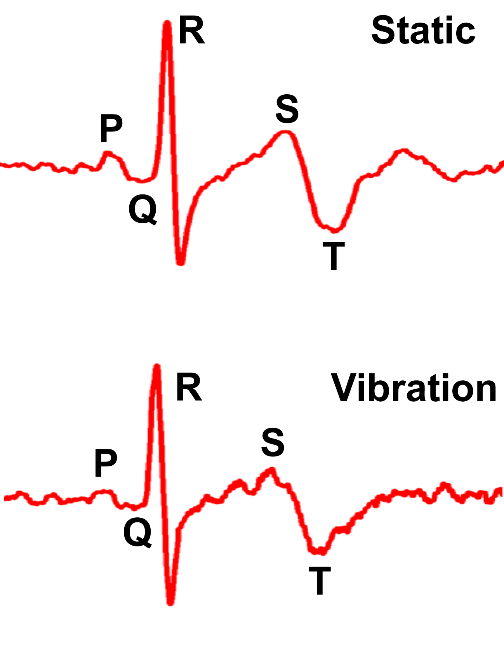


**Supplementary Figure 12** The characteristic peaks of ECG (P, Q, R, S, and T) were clearly distinguished by PTG.


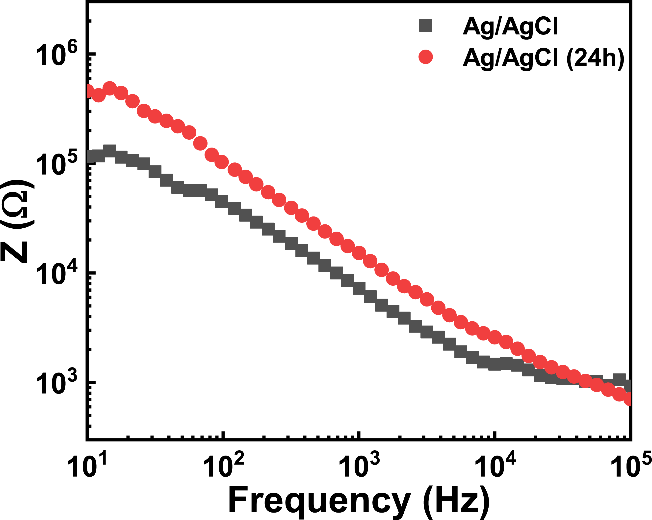


**Supplementary Figure 13** Dependence of contact impedance of Ag/AgCl gel electrodes on time. The contact impedance increased from 116 kΩ to 456 kΩ at 10Hz with gel getting dry.


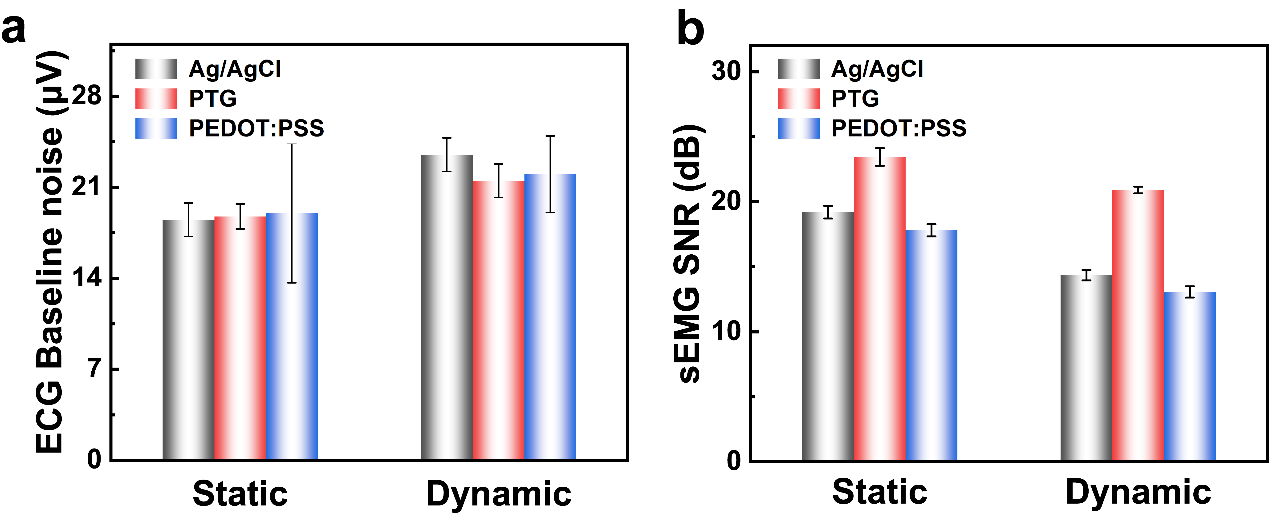


**Supplementary Figure 14** The baseline noise of ECG (a) and SNR of sEMG (b) measured by Ag/AgCl, PEDOT:PSS and PTG electrodes without and with vibration respectively. The signal-to-noise ratio (SNR) compares the level of signal to noise power, and is calculated as following: ${SNR}_{dB}=20lg(\frac{A_{signal}}{A_{noise}})$, where A is root mean square amplitude.


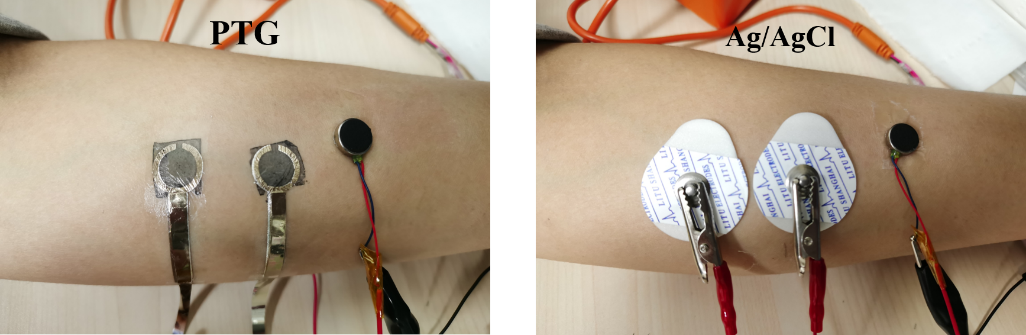


**Supplementary Figure 15** Evaluation of baseline noise in sEMG detection using an electromechanical vibrator to mimic skin vibration in arm movement. The electromechanical vibrator was placed near the working electrodes at an approximate distance of 2 cm. To stably connect PTG to the electrophysiological recording instrument, evaporated Ag films were aided in the connection between soft-hard interfaces.


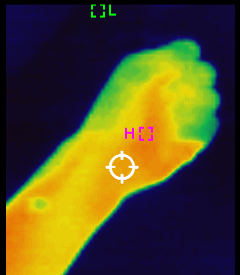


**Supplementary Figure 16** Infrared image of PTG on the forearm, showing that a minimal level of heat was accumulated upon mounting the PTG electrodes on a volunteer’s forearm.


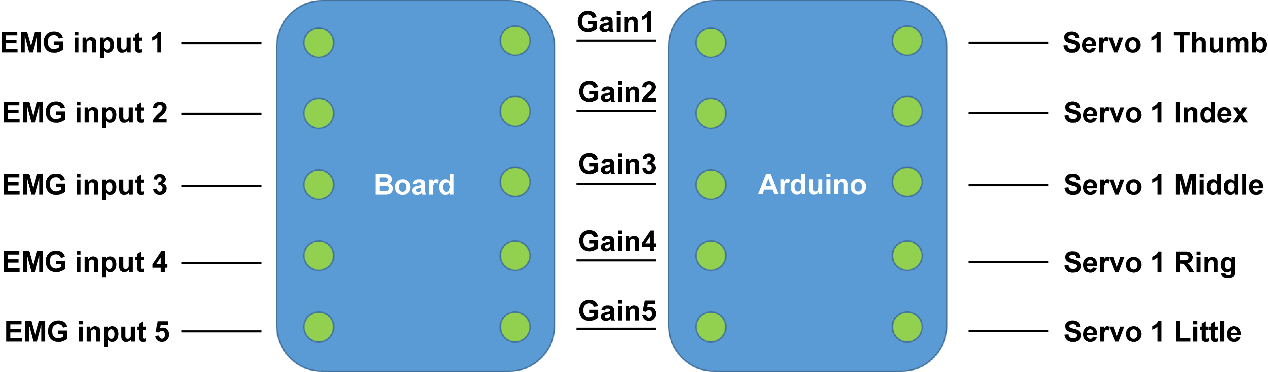


**Supplementary Figure 17** The overall functional block diagram. It includes front-end analog circuit acquisition and back-end digital signal filtering processing.


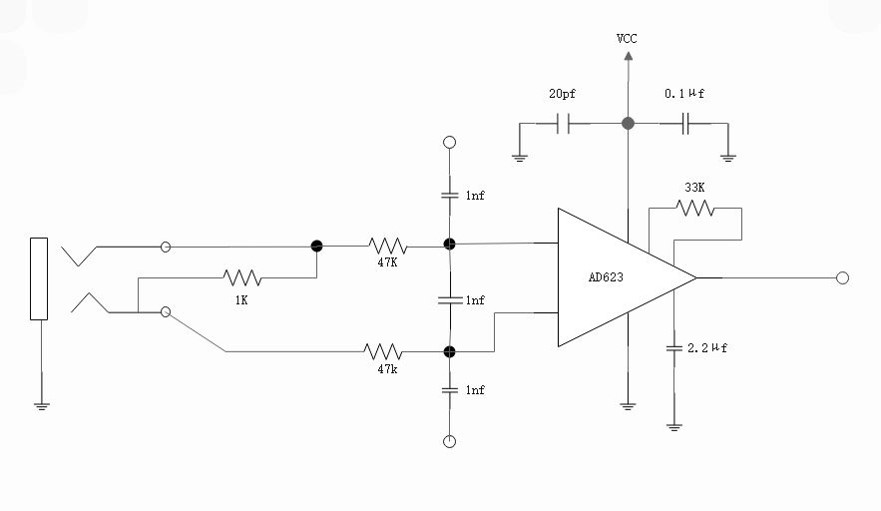


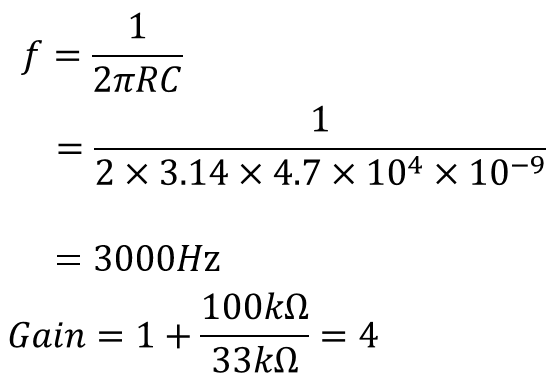


**Supplementary Figure 18** Circuit diagram of a low-pass filter (LPF).


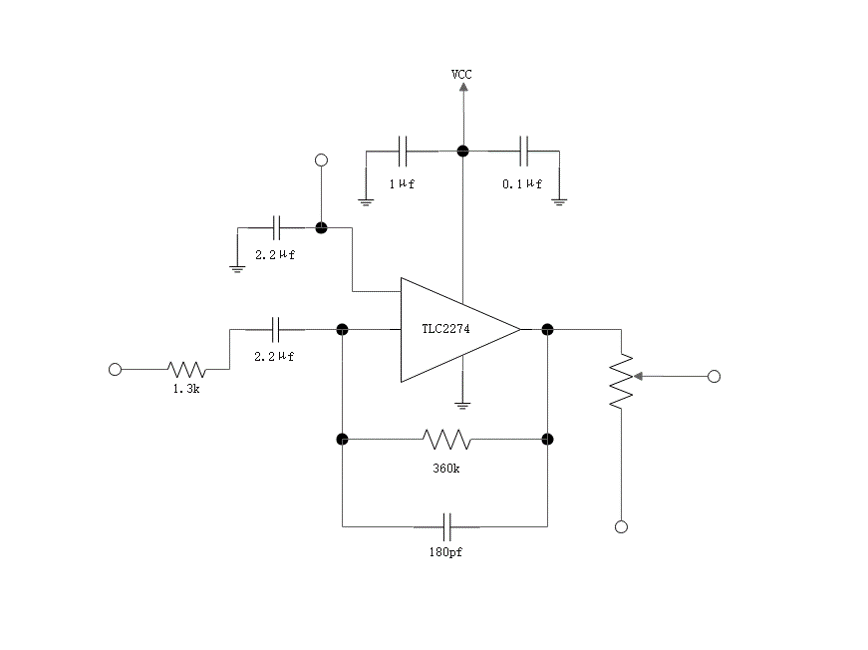


**Supplementary Figure 19** Circuit diagram of a filter, 55-2500 Hz with a gain of 177.


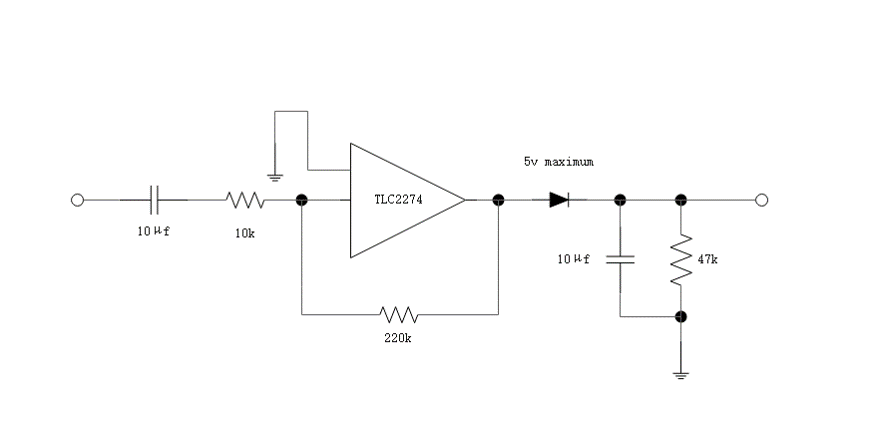


**Supplementary Figure 20** Circuit diagram of the envelope mode with a gain of 22.


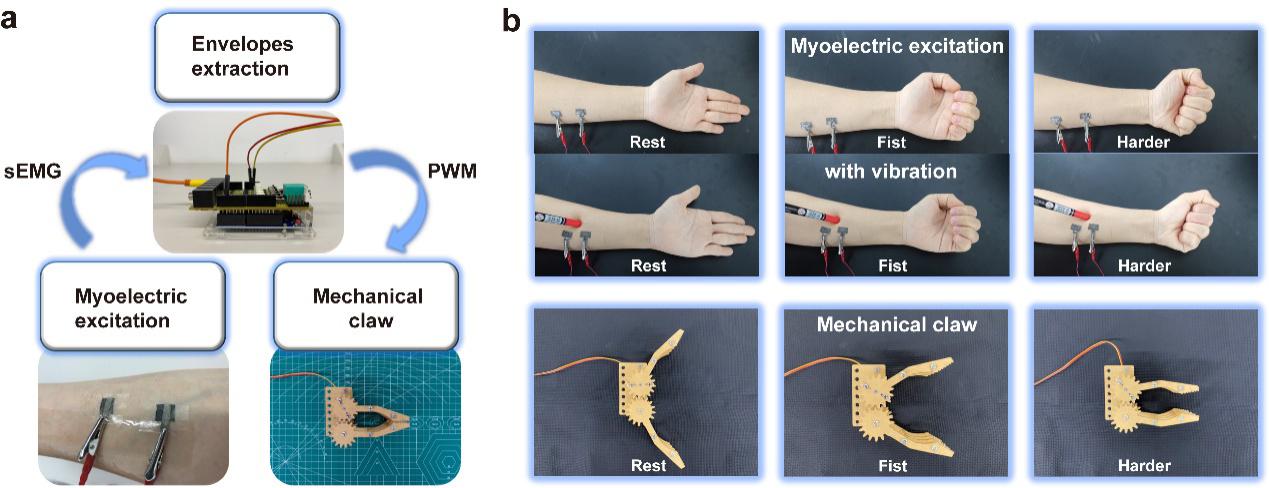


**Supplementary Figure 21** **a** Schematic illustration of PTG electrodes on the arm for sEMG detection and robotic claw controlling process. Firstly the sEMG signals were extracted by PTG electrodes and then converted to Pulse Width Modulation (PWM) pulses to drive the servo motor. **b** Demonstration of controlling a robotic claw during vibration. We knocked our arm via a pen to mimic skin vibration in dynamic state, and did not observe any interference while controlling the robotic claw.


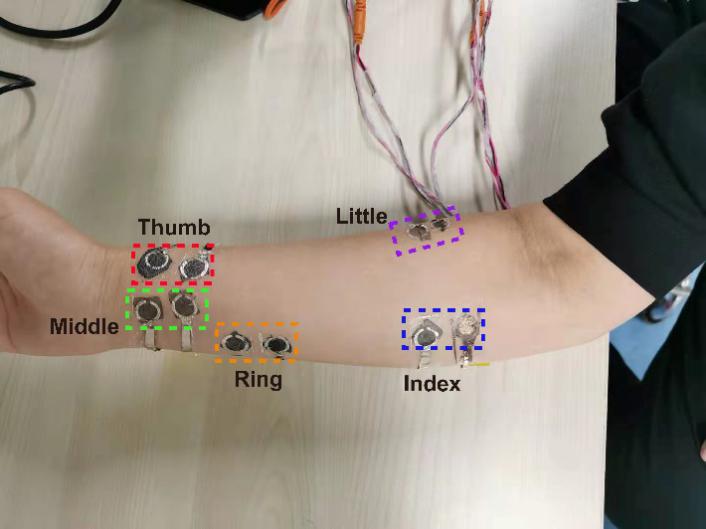


**Supplementary Figure 22** Five electrode pairs were adhered on designated locations to respectively control the movement of corresponding fingers of the robotic hand.


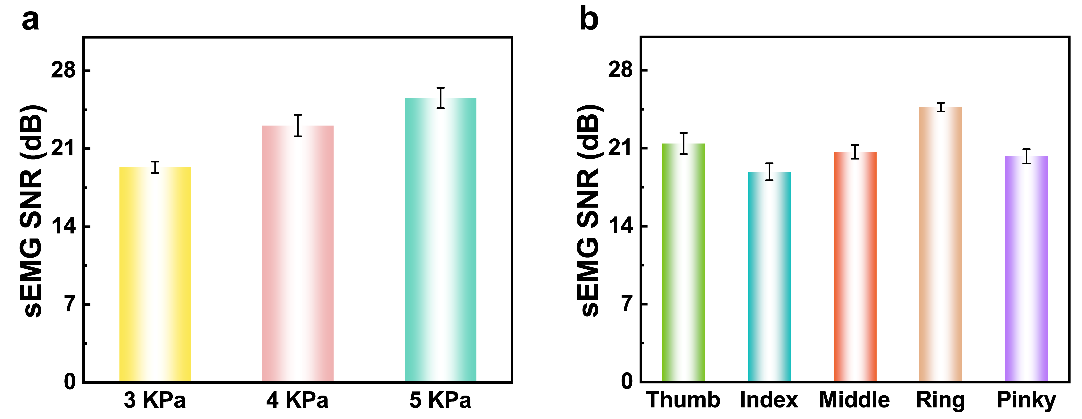


**Supplementary Figure 23** The SNR of sEMG generated with different griping force (a) and the SNR of sEMG generated by each finger (b).


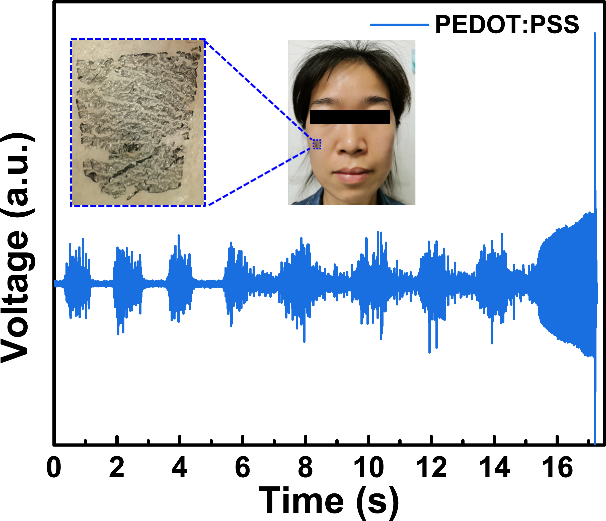


**Supplementary Figure 24** Facial sEMG measured by PEDOT:PSS only. With time going on, the movement of face is unavoidable (e.g.: smile, eating, talking…), leading PEDOT:PSS to be failed in sEMG measurement. Inset is a photo of the PEDOT:PSS dry electrode on face. After a short facial sEMG measurement, the PEDOT:PSS electrode is fractured and become insulative. Photo credit: Yan Zhao, State Key Laboratory of Digital Manufacturing Equipment and Technology, Beijing Normal University.


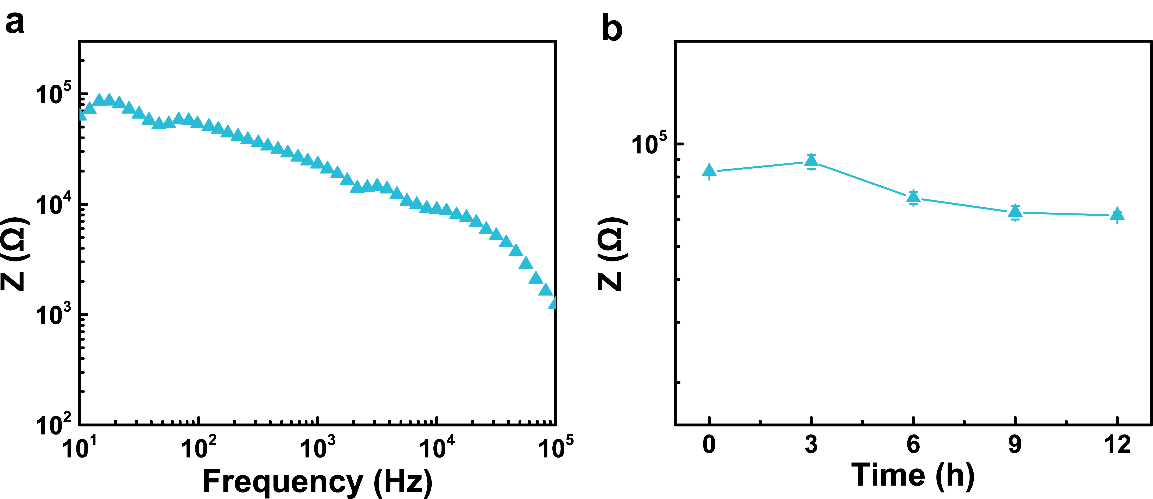


**Supplementary Figure 25** **a** Typical contact impedance of PTG on skin after 12 hours. **b** Contact impedance at 10 Hz as a function of time.

**Supplementary Table 1.** Comparison of the practical merits of various PEDOT:PSS electrodes for electrophysiology.

| **Methods** | **Material Properties** | **Signal Detection** | **ECG** | **sEMG (facial sEMG)** | **EEG** | **Human-machine interfaing** | **Ref.** |
| --- | --- | --- | --- | --- | --- | --- | --- |
| Inject-printed PEDOT:PSS | 520 Ω/sq (150 nm thick, transparent), conductive at 5% strain | ECG, sEMG | Similar SNR and noise voltage vs. Ag/AgCl in static status | Similar SNR and noise voltage vs. Ag/AgCl in static status | No EEG data | No | *Adv. Sci.* **5,** 1700771 (2018) |
| PWS | 545 S/cm (20 µm thick, opaque), stably conductive at 30% strain | ECG, sEMG, EEG | Lower noise voltage vs. Ag/AgCl both in static and dynamic status | No comparison with other electrodes (no facial sEMG data) | EEG at the stimuli of sound | One-channel | *Nat. Commun.* **11,** 4683 (2020) |
| PTG | 4142 S/cm (25 Ω/sq, 100 nm thick, transparent), conductive at 40% strain | ECG, EOG, sEMG, facial sEMG at largely deformed position, EEG | Higher SNR and lower noise voltage vs. Ag/AgCl both in static and dynamic status | Higher SNR and lower noise voltage vs. Ag/AgCl both in static and dynamic status | EEG monitoring for ~12 hours | Multi-channel | This work |

**Supplementary Table** **2.** Comparison of the optoelectronic properties of the state-of-the-art of graphene-based transparent electrodes.

| **Methods** | **R_sh_ (Ω/sq)** | **Transparency** | **References** |
| --- | --- | --- | --- |
| Solution-Processed rGO | 10^2^ -10^3^ | 80% | *ACS Nano* **2,** 463-470 (2008) |
| CVD graphene on Cu | 2100 (1 layer) | 98% | *Nano Lett.* **9,** 4359-4363 (2009) |
|  | 350 (4 layers) | 90% |  |
| CVD graphene on Ni | 280 | 80% | *Nature* **457,** 706-710 (2009) |
| Graphene/AgNWs hybrid film | 8 | 94% | *Nano Lett.* **15,** 4206-4213 (2015) |
| MGG | 185 | 88% | *Sci. Adv.* **3** e1700159 (2017) |
| GEF /monolayer graphene | 150 | 83% | *ACS Materials Lett.* **2,** 999-1007 (2020) |
| PTG | 45 | 80% | This work |

**Supplementary Table 3.** Comparison of the thickness and electrical conductivity of PTG and pure PEDOT:PSS films at various preparation conditions.

| Sample | Preparation condition | R_sh_ (Ω/sq) | Thickness (nm) | Conductivity (S/cm) |
| --- | --- | --- | --- | --- |
| Pure PEDOT:PSS | SDS 1.0 wt%+BSL 1.0 wt%, 4000 rpm | 70.1±0.2 | 83.5±2.2 | 1620 |
| Pure PEDOT:PSS | SDS 1.0 wt%+BSL 1.0 wt%, 3000 rpm | 80.2±0.4 | 185.0±4.1 | 674 |
| PTG (1G+PEDOT:PSS) | SDS 1.0 wt%+BSL 1.0 wt%, 3000 rpm | 43.8±0.9 | 79.7±3.7 | 2850 |
| PTG (1G+PEDOT:PSS) | SDS 1.0 wt%+BSL 5.0 wt%, 3000 rpm | 24.1±0.3 | 100±4.8 | 4142 |
| PTG (1G+PEDOT:PSS) | SDS 1.0 wt%+BSL 7.0 wt%, 3000 rpm | 14.6±0.7 | - | - |
| Graphene |  | 600±2 | ~1 |  |

Reference:

1. Taccola S. et al. Toward the Use of Temporary Tattoo Electrodes for Impedancemetric Respiration Monitoring and Other Electrophysiological Recordings on Skin. *Sensors* **21**, 1197 (2021).
2. Kireev D. et al. Multipurpose and Reusable Ultrathin Electronic Tattoos Based on PtSe2 and PtTe2. *ACS Nano* **15**, 2800-2811 (2021).
