# Supplementary material for: Ultra-conformal skin electrodes with synergistically enhanced conductivity for long-time and low-motion artifact epidermal electrophysiology
Source: Nat Commun. 2021 Aug 12;12:4880. doi: 10.1038/s41467-021-25152-y (PMC8361161; doi:10.1038/s41467-021-25152-y)
Supplement: Supplementary file 3 — Description of Additional Supplementary Files [file 41467_2021_25152_MOESM3_ESM.docx]

Supplementary Movie 1. Remotely controlling the robotic hand to show gestures of victory and holding a fist based on multi-channel sEMG signals extracted by PTG electrodes on thumb, index, middle, ring and little fingers.

Supplementary Movie 2. Remotely controlling the robotic hand to show gestures of victory and rotating the wrist based on multi-channel facial sEMG signals extracted by PTG electrodes on zygomaticus, risorius and corrugator.
